# Phylogenetically typing bacterial strains from partial SNP genotypes observed from direct sequencing of clinical specimen metagenomic data

**DOI:** 10.1186/s13073-015-0176-9

**Published:** 2015-06-09

**Authors:** Jason W. Sahl, James M. Schupp, David A. Rasko, Rebecca E. Colman, Jeffrey T. Foster, Paul Keim

**Affiliations:** Department of Pathogen Genomics, Translational Genomics Research Institute, Flagstaff, AZ USA; Center for Microbial Genetics and Genomics, Northern Arizona University, Flagstaff, AZ 86011 USA; Institute for Genome Sciences, University of Maryland School of Medicine, Baltimore, MD USA; Current address: Department of Molecular, Cellular & Biomedical Sciences, University of New Hampshire, Durham, NH USA

## Abstract

We describe an approach for genotyping bacterial strains from low coverage genome datasets, including metagenomic data from complex samples. Sequence reads from unknown samples are aligned to a reference genome where the allele states of known SNPs are determined. The Whole Genome Focused Array SNP Typing (*WG-FAST*) pipeline can identify unknown strains with much less read data than is needed for genome assembly. To test *WG-FAST*, we resampled SNPs from real samples to understand the relationship between low coverage metagenomic data and accurate phylogenetic placement. *WG-FAST* can be downloaded from https://github.com/jasonsahl/wgfast.

**Electronic supplementary material:**

The online version of this article (doi:10.1186/s13073-015-0176-9) contains supplementary material, which is available to authorized users.

## Background

Whole genome sequencing (WGS) is a powerful and increasingly available technology for understanding the evolutionary and epidemiological relationships among bacterial pathogens. For bacterial disease outbreaks, whole genome analysis has been used to identify and attribute the outbreak sources for many bacterial pathogens, including *Escherichia coli* O104 [1], *Vibrio cholerae* [2], *Klebsiella* spp. [3], methicillin resistant *Staphylococcus aureus* (MRSA) [4] and even *Bacillus anthracis* [5]. Adding the genetic relationships of isolates to other standard epidemiological correlates (for example, time and space) offers the power to identify disease outbreaks that would not otherwise be apparent. This approach has been highly successful using sub-genomic DNA methods (for example, multi-locus sequence typing (MLST) [6]) but the use of whole genome sequencing will replace these in the near future due to precision and accuracy of strain identification offered by this near comprehensive technology [7].

The advent of molecular diagnostics (for example, polymerase chain reaction (PCR)) has led to improved pathogen identification, in part, because they are not dependent upon isolation and subsequent culturing of the pathogen. But the currently dominant disease-tracking methods (for example, pulsed field gel electrophoresis (PFGE)) only work with isolated pure cultures, leading to the possibility that disease tracking efforts will be diminished in this new age [8]. Molecular epidemiological methods using the power of WGS that parallel molecular diagnostics with direct application to complex specimens are needed. In fact, recent studies have used this approach to associate diseases with the infectious agent [9, 10].

WGS analysis to identify pathogen strains would seem possible through the metagenomic deep sequencing of clinical specimens, but genome coverage of a specific microbe is hard to predict and the pathogen may represent only a minor component in the microbiome of the infected tissue [11]. Many pathogen populations have low diversity and, hence, single nucleotide polymorphism (SNP) discovery with low-genome coverage leads to greater misidentification due to sequencing errors than true SNP genotyping. To reduce this ‘signal-to-noise’ problem, we developed the Whole Genome Focused Array SNP Typing (*WG-FAST*) method, where only known SNPs with defined allele states are scored. These are derived from a reference population where high quality genomic data are available to generate a highly robust phylogenetic reconstruction. Sequencing reads are aligned to a reference genome annotated with the positions of known SNPs and their allelic states. The metagenomic SNP genotype of the unknown pathogen can then be placed into the most likely phylogenetic position. It is the reference population SNP database that defines the best possible model for population structure, which is then used as a reference for unknown SNP genotypes identified from less than ideal (for example, low coverage) datasets. We also present several approaches for establishing confidence in phylogenetic placement including hypothesis-testing methods that generate odds ratio probabilities. This is essential because the precision of phylogenetic placement will be unique for each application and is dependent upon a number of variables including: (1) the SNP/genome density in the reference population; (2) the depth of genome coverage from the unknown sample; and (3) the phylogenetic topology in the actual placement position of the reference population. Thus, placement confidence metrics must be established for each unknown sample. *WG-FAST* will allow the use of deep metagenomic sequencing data to identify strains from complex samples such as clinical specimens, food matrices, and the environment, alleviating the requirement for pure cultures to accomplish molecular epidemiological goals.

## Methods

### Single nucleotide polymorphism (SNP) discovery

The robust characterization of SNPs in a reference set of isolates is a necessary first step in the *WG-FAST* analysis pipeline. A pipeline to wrap methods discussed below, known as the Northern Arizona SNP Pipeline (NASP), is publically available (tgennorth.github.io/NASP/). Our strategy for reference SNP identification is to use only the non-redundant core genome sequences to avoid missing data and misuse of paralogous regions. To create a reference database, raw reads or assembled genomes are aligned to a reference genome with BWA-MEM [12] or NUCmer [13], respectively. SNPs and insertion/deletions (indels) can be identified with variant callers including the UnifiedGenotyper method in GATK [14, 15], SAMtools [16], VarScan [17], and/or SOLSNP ([18]). Called SNPs can then filtered using user-defined thresholds for read depth (default = 3×) and allele frequency proportion (default = 90 %). All called SNPs are then placed into a matrix that includes the nucleotide calls in each position of the reference genome for all genomes queried. Benchmarking tests on a single genome (*E. coli* C227-11) with 12 million reads, 100 bases in length, took 4 h 25 min to place and perform 100 subsampling confidence tests using eight processors on a single node with 48 Gb of RAM.

### Whole genome focused array SNP typing (*WG-FAST*) pipeline

Source code for *WG-FAST* is publically available at [19] under a GPL v3 license. The required input for a *WG-FAST* analysis includes a NASP-formatted SNP matrix, a phylogeny inferred with RAxML [20], a reference genome assembly, and a directory including single or paired-end reads with ‘.fastq.gz’ extensions. Dependencies for *WG-FAST* include BWA-MEM, GATK, Picard-tools ([21]), DendroPy [22], RAxML v8 [23], BioPython [24], Trimmomatic [25], and SAMtools [16]; many of these dependencies are included in the *WG-FAST* repository. A script to generate the formatted, required phylogeny from the SNP matrix is included with *WG-FAST*.

**Fig. 1:**
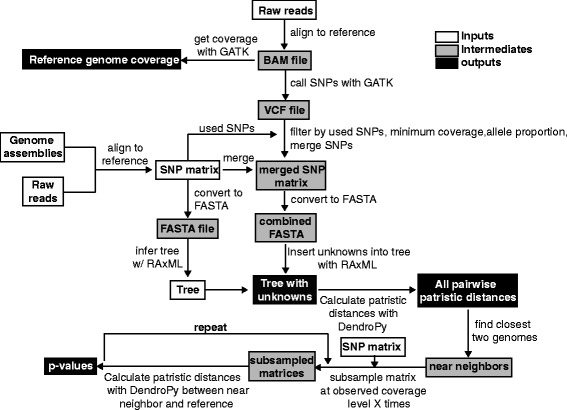
A workflow of the *WG-FAST* pipeline. Input files are shown in white boxes and include a SNP matrix, a corresponding phylogeny, a reference genome assembly, and a directory of reads from all unknowns. The output files include a phylogeny with unknowns highlighted in red, all pairwise patristic distances, the average depth of coverage across the reference assembly, and if the subsample routine is invoked, the resulting *P* values

In the *WG-FAST* pipeline workflow, reads are initially mapped to a reference genome assembly with BWA-MEM and SNPs are called with the UnifiedGenotyper method in GATK. The resulting variant call format (VCF) file is then filtered for minimum coverage and minimum allele proportion. If a position fails a filter, then the call is replaced with a gap (‘-’), indicating missing data. The VCF file is also filtered to only include genomic coordinates present in the input SNP matrix. The unknowns are then merged with the original SNP matrix, which is converted into a multi-FASTA file. All unknowns are then inserted into the phylogenetic tree using an evolutionary placement algorithm (EPA) method in RAxML [26]; this method assigns unknowns to edges of the phylogeny based on a maximum likelihood algorithm. When the final tree is opened with FigTree ([27]), all unknown genomes are displayed in red for easy visualization. All patristic distances are calculated with DendroPy [22], and the most closely related genomes to each unknown, based on the lowest patristic distance, is identified and reported. A schematic of the complete *WG-FAST* pipeline is shown in Fig. 1.

An optional subsampling routine is built into *WG-FAST* in order to test the robustness of a given placement on a phylogenetic tree. From the final phylogeny, the two closest genomes to each unknown, based on patristic distances, are identified. The SNPs from the two neighbors are then sampled at the same coverage level as each unknown and a new SNP matrix is created. Each matrix is then converted into a multi-FASTA and the samples are placed into the phylogeny with the EPA algorithm. The patristic distance to the reference isolate is then calculated for each subsample and is compared to the ‘true’ patristic distance using all SNPs; the reference is used because its position is fixed and the ‘Reference’ name is the same for each NASP-formatted SNP matrix, regardless of the target organism. The null hypothesis is that a random subsampling placement will differ significantly from the ‘correct’ placement based on a comparison of patristic distances. The number of times that the distance from the reference is different from the known placement is calculated for 100 replicates based on a user-defined threshold. From a set of 100 replicates, if the number of samples placed incorrectly is fewer than 5, then the *P* value is <0.05 and the placement can tentatively be trusted. For large datasets (that is, hundreds of thousands of SNPs and hundreds to thousands of genomes), this subsampling routine may be impractical, as 200 placements are required if 100 replicates is selected by the user.

### A *WG-FAST* test case

To test the utility of the *WG-FAST* pipeline, approximately 700 *E. coli* genome assemblies were downloaded from GenBank [28]; *E. coli* was used as the test case due to the large number of assembled genomes in public databases and due to the non-clonal nature of the species [29]. SNPs from all genomes were identified using *E. coli* K-12 W3110 (accession # NC_007779) [30] as the reference, and a SNP matrix was generated with NASP. A maximum likelihood phylogeny (Additional file 1: Data file 1) was inferred on this concatenated SNP alignment with RAxML v. 8.1.13 using the following parameters: −f d -p 12345 -m GTRGAMMA. Closely related genomes, based on phylogenetic relatedness, were then manually removed, resulting in 255 genomes (Additional file 2). Autapomorphic SNPs (that is, private SNP alleles) in the outgroup genome, TW10509 (AEKA00000000) [31], belonging to a ‘cryptic’ lineage of *E. coli* [32], were also removed. The resulting SNP matrix consisted of greater than 225,000 SNPs (Additional file 3: Data file 2).

### Read subsampling

To test the robustness of the *WG-FAST* pipeline using a low number of reads, sequence reads were randomly sampled at varied depths (50–100,000 read pairs), from published *E. coli* datasets (Additional file 4). One hundred separate datasets at each read depth were then processed with *WG-FAST*. The minimum number of called positions in order to correctly genotype the unknown ≥95 % of the time, based on a patristic distance ratio (query patristic distance to reference/true patristic distance to reference) between 0.99 and 1.01, was identified for each genome. Multiple isolates from different regions of the tree and sequence data from multiple sequencing platforms were analyzed.

### SNP subsampling

In addition to subsampling raw reads, positions present in the SNP matrix were subsampled for each genome in the phylogeny. SNPs were sampled at a lowest frequency of 50, then sampled every 100 SNPs subsequently, until the patristic distance of 95 % of 100 iterations, compared to the reference, was between 0.99 and 1.01, compared to the patristic distance of the placement using all available positions.

### SNP matrix correlation with subsamplings

To identify the fewest number of reference positions required in order to obtain a comparable matrix to a matrix using all available SNPs, a subsampling method was employed. A user-provided number of SNPs were randomly selected from the matrix, the reduced matrix was converted into a multi-FASTA, and a distance matrix was calculated with mothur [33]. A distance matrix was also generated from the complete SNP matrix with mothur. A Mantel test was then performed on the two matrices with mothur, using the Pearson correlation. The Pearson correlation value at each SNP level, with 100 replicates, was then plotted. A script to wrap these functions is available with *WG-FAST* (subsample_snps_pearson.py).

### Metagenomic analysis

To test the *WG-FAST* method on metagenomic samples, 53 datasets from a recent metagenomic survey of stool samples from the 2011 *E. coli* O104:H4 outbreak [34] were downloaded and processed with *WG-FAST* (Additional file 5). The subsample routine was run on all samples using 100 iterations.

### *In silico* mixtures

In some clinical samples, mixtures of multiple closely related conspecific strains have been observed [35]. To determine how mixtures will affect phylogenetic placement using *WG-FAST*, several artificial mixtures were generated (Additional file 6) and processed with *WG-FAST*. When processed with *WG-FAST*, a minimum coverage of 1× and a minimum proportion of 60 % was used.

### Error rate calculation

*WG-FAST* is intended to phylogenetically genotype isolates from complex samples where the desired signal could be faint. In these cases, error in the data could confound accurate phylogenetic characterization. To test the error rate, raw reads were mapped against the *E. coli* genome TY-2482 (SRR292862) with BWA-MEM, and a BAM file was generated. At each position in the reference chromosome, the most frequent base was removed and the counts of the alternate alleles, which represents error, were summed. Average error and associated standard deviation was calculated across the entire reference chromosome.

## Results

### Whole genome focused array SNP typing (*WG-FAST*) pipeline

*WG-FAST* was developed as a parallel, open source method to accurately genotype novel isolates from high read coverage (for example, 50× reference genome coverage) or from metagenomic data in the context of a known phylogenetic or population genetic structure (Fig. 1). This method can be used to type new bacterial populations, where the tree structure should either not be altered, the read depth is low (<1×), as is the case with metagenomic samples, or where computation of a new tree is too computationally expensive. *WG-FAST* is an open-source application written in Python and relies on published and validated tools for read alignment, single nucleotide polymorphism (SNP) calling, and the placement of samples in a phylogenetic context.

### Intrinsic error rate

One potential pitfall to identifying SNPs from low coverage samples is mistaking sequencing errors for true variants. We estimated the single-read base call error rate across the *E. coli* chromosome in isolate TY-2482 [36] to understand its effect on genotyping accuracy; the average error rate in this dataset was 0.16 % (SD ± 0.43 %) (Additional file 7). Although this error rate is low, at 1× coverage of a model bacterial genome (for example, 5 Mbp) this would result in the discovery of roughly 8,000 false SNPs. These errors would lead to incorrect calls across the reference genome, which would confound the analysis of true SNPs in many epidemiological analyses where the true variation can be much less. While the use of short read error correction tools, such as Hammer [37] or Musket [38], prior to *WG-FAST* should reduce many of these errors, the common solution is to increase sequence coverage to verify a particular SNP. With high read coverage, the false SNP discovery is small, but this is difficult and expensive to achieve in a metagenomic analysis of complex specimens. Rather, the *WG-FAST* approach limits base calling to known SNP positions and therefore minimizes the impact of this error rate. In a 1,000 SNP genotype, fewer than 2 SNPs would be falsely identified at this rate. If metagenomic data are used to generate a genotype at known genomic positions and with known allele states, the sequencing error has little consequence on a multi-locus genotype determination.

### *Escherichia coli* dataset and phylogeny

As a *WG-FAST* test case, approximately 700 *E. coli* genome assemblies were downloaded from GenBank; *E. coli* was used as a test case due to the large number of sequenced genomes. For this analysis, closely related genomes were manually removed based on phylogenetic redundancy, resulting in a dataset of 255 genomes (Additional file 2). SNPs were then identified from NUCmer [39] alignments and a phylogeny was generated from the concatenated SNP alignment (approximately 225,000 SNPs) with RAxML v8 [23], using TW10509 (accession #AEKA00000000) as the root (Additional file 8) and K-12 W3110 [30] as the reference. The retention index (RI) [40] of the tree was 0.80, demonstrating significant homoplasy in the underlying SNP data, probably resulting from historical recombination among lineages. However, the major *E. coli* phylogroups, labeled A through E, were monophyletic and consistent with previous analyses [41].

### Subsample SNP correlations

**Fig. 2:**
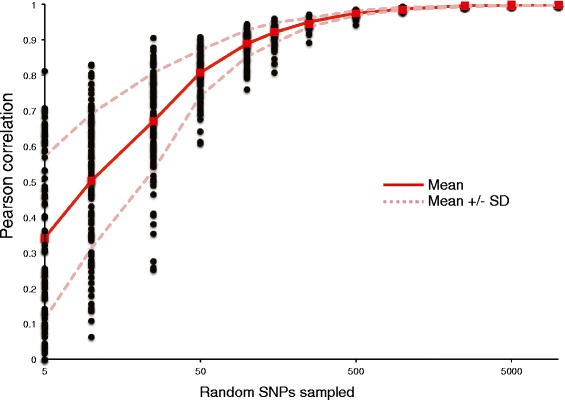
The effect of increasing SNP loci on genetic distance estimation. A pairwise distance matrix between 255 *E. coli* genomes and based upon approximately 225,000 SNPs was calculated and then correlated to matrices generated using fewer SNPs. SNP subsampling was performed 100 times at each level. Each subsampled matrix was then converted into a multi-FASTA and a distance matrix was calculated with mothur [33]. Distance matrices were compared with the Mantel test function in mothur and the Pearson correlation value was calculated and plotted for each subsampling level. The mean and standard deviation for all iterations were calculated and plotted

At a gross level, population genetic structure is frequently estimated by calculating a pairwise distance matrix among all the genomes in a study. In order to understand how many SNPs are needed to accurately establish the population distance structure, we subsampled the SNP matrix at different levels and calculated a pairwise distance matrix. We then correlated the resampled data to the original distance matrix using a Mantel test with the Pearson correlation; a script to perform these functions is included with *WG-FAST*. The subsampled distance matrices had a poor correlation to the complete matrix and high variance when only 5 SNPs were used to calculate genetic distance, but the correlation increased rapidly with increasing numbers of SNPs (Fig. 2). The results demonstrate that at 500 SNPs, there is strong correlation (>0.9) between the original distance matrix and the subsampled matrix. The correlations only slightly improved with greater than 500 SNPs. While using all available data is prudent, this demonstrates that many fewer than the full 225,000 SNPs are needed to accurately estimate the relationships in this reference population. Nevertheless, precise phylogenetic placement is more than a simple genetic correlation and must be explored with more detailed methods.

### Subsampling SNPs from the complete SNP matrix

**Fig. 3:**
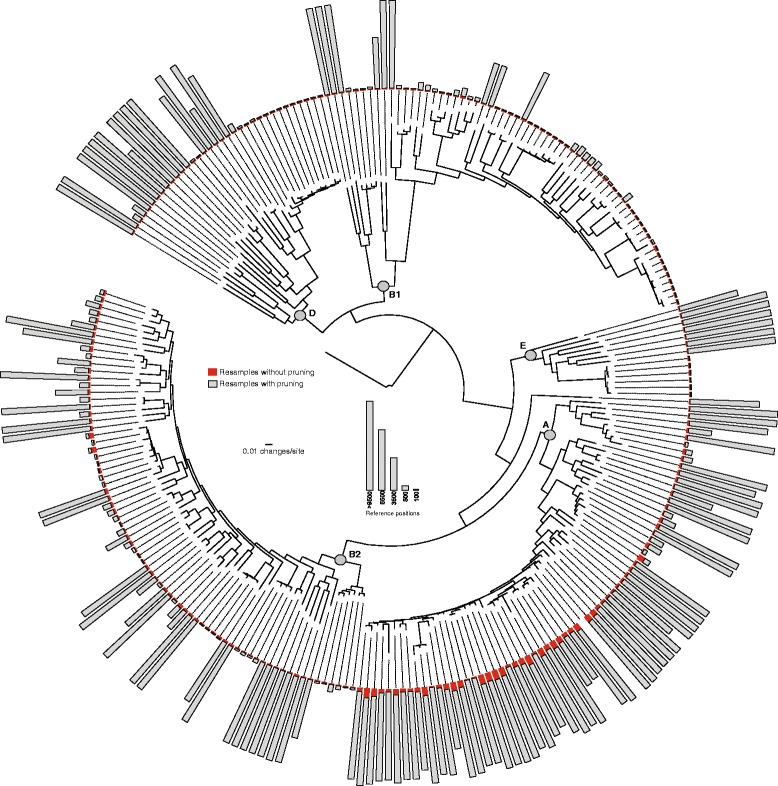
The effect of SNP number on phylogenetic placement. The SNPs were randomly subsampled from each genome at different levels and were then inserted into the phylogeny 30 separate times with either the genome removed (gray) or not removed (red). The number of reference positions required for accurate placement are illustrated by the height of bars, but were capped at 9,500 positions. The phylogenomic clade designations (a-e) are shown at each relevant node

In order to understand the consequence of the population structure on *WG-FAST*, we subsampled SNPs for all 255 genomes and examined the accuracy of phylogenetic placement. SNPs were subsampled from the matrix for each genome at different depths and the genome was then re-inserted into the phylogeny. In the first iteration, the queried genome was not pruned from the reference tree, which provided a precise target for subsequent placement. Using this method, as few as 100 positions could result in accurate placement >95 % of the time (Fig. 3), although variation was observed across clades, as well as for individual genomes. In the next iteration, each genome in the reference phylogeny was subsampled at different depths and pruned from the phylogeny. The subsampled genome was then re-inserted into the phylogeny with RAxML and the results tabulated (Fig. 3). In general, the lack of a precise target required more SNP loci for accurate phylogenetic placement. In some phylogenetic positions this was dramatically different, though not universal as some clades and positions had very similar accuracy between the two iterations. The results demonstrate that if the exact or a closely related genome is in the phylogeny, far fewer SNPs are required for accurate placement than if the genome represents a new node or branch in the phylogeny. Thus, the precision of the *WG-FAST* approach is highly dependent upon the reference phylogeny and even the position within the phylogeny, with well-sampled clades giving higher genotyping resolution. This argues for the generation of large population reference sets with an emphasis on clinically important strains to increase the probability of a near or perfect match. Regardless, resampling of the underlying SNPs for any reference phylogeny can be used to understand assignment power at each position and more SNPs will be needed for accurate placement at some positions than for others.

### Subsampling Reads for *WG-FAST* placement

**Fig. 4:**
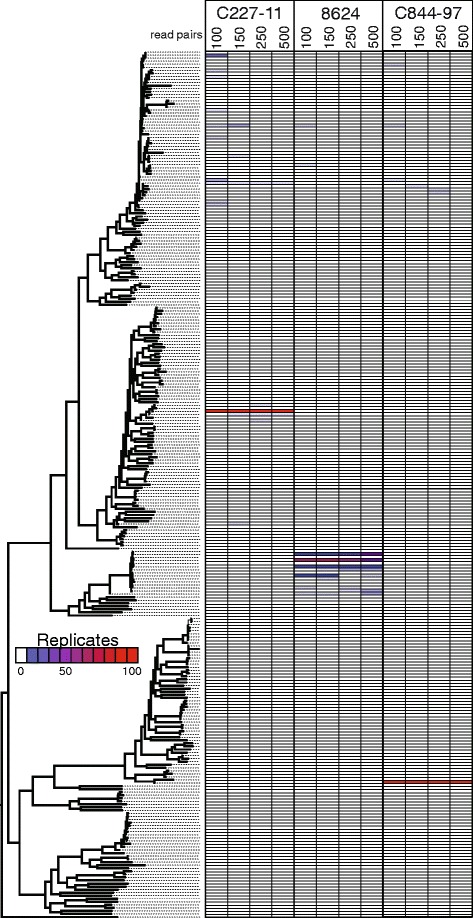
A phylogeny and associated heatmap showing the placement of read subsamples for 100 iterations of three *E. coli* isolates. The maximum likelihood phylogeny was inferred by RAxML [20] from a concatenation of approximately 225,000 single nucleotide polymorphisms called against the reference genome, K-12 W3110 [30]. Raw reads were randomly sampled from three genomes at four different depths and reference positions were identified. Each genome, at each level, was then inserted into the phylogeny with the evolutionary placement algorithm (EPA) in RAxML [26]. Duplicate placements were removed from the tree and redundancies were represented as a heatmap

*WG-FAST* was designed for metagenomic datasets where the number of reads mapping to a reference genome will be variable and difficult to control or predict. The accuracy of phylogenetic placement will be greater with more reads, but also dependent upon whether these reads align to known SNP positions to allow determination of the allelic state. We have resampled between 200 and 2,000 raw reads from different genomes from each major group from the phylogeny (Additional file 4), aligned them to the reference genome, and then determined the SNP allele states for positions with mapped reads. These limited genotypic data were resampled 100 times each and then placed with RAxML onto the reference tree and the frequency of placement represented as a heatmap (Fig. 4); the patristic distances of all subsamples compared to the ‘correct’ placement demonstrates that additional SNP loci genotyped increases the quality of the placement (Additional file 9). The accuracy of placement increases with larger read resamples, but as with SNP resampling (Fig. 3), this relationship changes based on phylogenetic position. Clades with many closely related genomes complicate exact positioning of an unknown, especially with smaller read datasets, though near-misses are very common and might be sufficient for some studies. As one demonstration of the potential, however, we demonstrate that the O157:H7 Sakai genome could be accurately placed on the tree >95 % of the time with as few as 360 SNP loci genotyped from only 50 Illumina MiSeq (2 × 250 bp) read pair alignments (Additional file 4).

### Strain mixtures

In some cases, a single pathogen from a given species will be dominant in a clinical specimen [42], but not always. To test the effect of strain mixtures *in silico*, we used *E. coli* as the test case, which is a normal inhabitant of the healthy human gut. Reads from the reference isolate O104:H4, strain C227-11 [1], were mixed with reads from the O157:H7 isolate 8624 [43] at different proportions (90:10, 80:20, 70:30, 60:40) in a total of 10,000 read pairs (100 bp reads) (Additional file 6). At a read mixture of 80:20, the dominant sample was still accurately genotyped, although a longer branch length was observed due to homoplasious SNPs and unwarranted additional phylogenetic steps (Additional file 10). At a 70:30 mixture, the unknowns were no longer placed into the dominant strain clade, and could not be accurately typed. At a 60:40 mixture, most samples erroneously grouped with the reference with longer branch lengths. Strain mixtures at near equal proportions, which is not anticipated based on analyses of stool samples, would definitely confound accurate placement with *WG-FAST.* Importantly, however, this problem can be identified due to the presence of long branches leading to each unknown sample with highly homoplasious characters. More detailed analyses to identify the homoplasious SNPs and separate them has the potential to deconvolute mixtures into the source genotypes and allow their phylogenetic placement.

### Metagenomic sample analysis

**Fig. 5:**
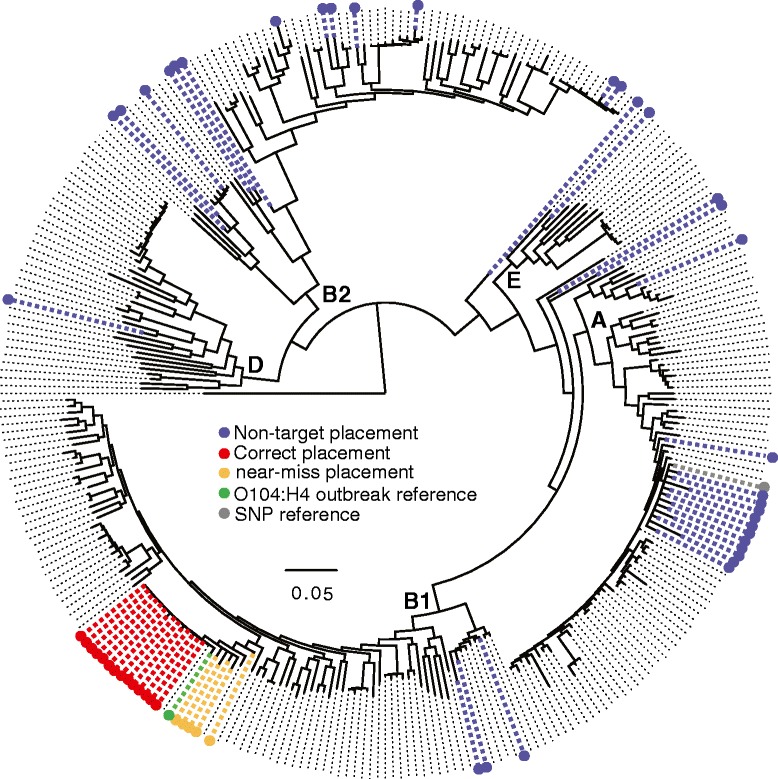
A phylogenetic tree showing the placement of 53 metagenomic samples associated with the 2011 outbreak of *E. coli* O104:H4 [34]. Raw reads from each sample were mapped against the reference genome, K-12 W3110 [30], and all positions were called by GATK [15]. Filtered positions were then inserted into the *E. coli* phylogeny with the evolutionary placement algorithm (EPA) in RAxML [26]. The placement of the reference O104:H4 isolate, TY-2482, is shown in green, the reference genome is shown in gray, correctly genotyped samples are shown in red, while other genotyped *E. coli* isolates are shown in blue

Metagenomic sequences of fecal specimens (53 datasets from 45 separate individuals) were generated during the investigation of the enteroaggregative/Shiga-toxin producing *E. coli* O104:H4 foodborne disease outbreak [34] and were processed in this report with *WG-FAST*. In a previous study using a separate informatics pipeline, the authors identified 12 of 53 stool samples as containing O104:H4 sequence and appeared to not be mixed with other *E. coli* genotypes, although several of the calls, based on coverage of MLST markers, were partial. In another seven samples they found that there were *E. coli* mixtures including O104:H4. *WG-FAST* correctly genotyped all 12 un-mixed samples as O104:H4 and also correctly classified the pathogen in four of the seven samples reported as mixed (Additional file 5 and Fig. 5); for three of the samples (2772-H, 2880-H, 4168-H), *WG-FAST* reported the sample as a near miss to the target, but were reported as not determined by the authors. This demonstrates the ability of *WG-FAST* to use low numbers of sequence data to accurately genotype samples, where other methods may require additional data.

### Metrics to measure placement accuracy

**Fig. 6:**
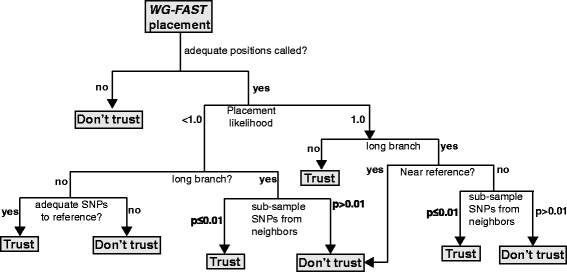
A decision tree to help a user decide the confidence in the *WG-FAST* placement of a sample

Several methods have been provided with *WG-FAST* to help a user assess the robustness of a phylogenetic placement. The first piece of evidence is if an unknown meets the minimum number of SNP positions for accurate placement, which will ultimately depend on the dataset analyzed. Scripts are included with *WG-FAST* that can be used to identify the number of required positions across a phylogeny for robust phylogenetic placement. Additionally, the EPA algorithm in RAxML [26] produces a file that contains the insertion likelihood for each placement. If the insertion likelihood is 1.0, the placement is reflective of the input data; the insertion likelihood values generally scale with the number of SNPs kept in a dataset (Additional file 11). To further quality check a placement, an optional subsample routine is included with *WG-FAST* that identifies the two closest genomes to the unknown, based on patristic distances, prunes them from the phylogeny, then subsamples the two neighbors at the same number of called positions as the unknown and re-inserts those genomes back into the phylogeny. The patristic distances between the two near neighbors and the reference are compared to the subsamples and the reference to see how SNP subsampling affects placement. A placement *P* value is then calculated by dividing all placements by the number of correct placements, based on a user-defined threshold, for each near neighbor sampling. The subsampling approach demonstrates that greater sequencing depth is correlated with *P* value significance (Additional file 12). A decision tree is provided to give a user confidence in a placement, based on several criteria (Fig. 6).

## Discussion

Analysis of the microbiome has been largely performed at higher taxonomic levels (for example, genus, species) and focused primarily upon the 16S rRNA gene [44] but these analyses are now increasingly using full metagenomic data sets [45]. This offers the opportunity to move the taxonomic discrimination to levels below that of species (that is, precise strain identification). However, genome-based classification is still complicated by low coverage datasets and ambiguous classification due to database biases, including incomplete datasets for many organisms. For pathogen identification, multiple reference genomes are usually available for a known pathogen and should only increase as whole genome sequence (WGS) data becomes easier to generate and analyze.

The availability of WGS data has led to the problem of how to analyze newly sequenced isolates in the context of existent data. This is especially a problem when trying to accurately genotype the causative agent of infections from WGS obtained directly from clinical specimens. Our *WG-FAST* pipeline is able to phylogenetically genotype isolates from single isolate sequencing projects, low coverage sequencing projects, or from complex samples with variable coverage, such as metagenomics projects sequenced from human samples. Specifically, *WG-FAST* was designed to accurately genotype new isolates where the read coverage is below 1× and may not be able to be genotyped by methods that rely on higher query coverage. Although studies have been published that use a similar concept [46], *WG-FAST* represents the only publically-available pipeline that can perform these functions and provide statistical support for a given phylogenetic placement. Although *WG-FAST* currently only works on SNP data, other data, including indels, can also be used to discriminate between related isolates and may provide additional phylogenetic resolution between genomes.

To test the application of *WG-FAST* towards real samples, a set of 53 metagenomic datasets from an analysis of diarrheal samples associated with a *Escherichia coli* O104:H4 outbreak [34], were processed. All samples identified by Loman *et al.* as positive for O104:H4 and unmixed, based on a separate bioinformatics pipeline, were also correctly genotyped by *WG-FAST*. However, additional samples were identified as positive for O104:H4 by *WG-FAST* that were reported as negative by Loman *et al.* This demonstrates that the ability of *WG-FAST* to genotype based on partial genotypes may allow for lower level detection than read assembly or mapping methods that require higher reference coverage in order to classify a pathogen. The long branches on some samples demonstrate signs of mixtures of multiple isolates (Fig. 5), which were also identified by Loman *et al.*; because *WG-FAST* does not discover novel SNPs, any branch lengths are indicative of homoplasy created by character state conflicts.

The placement of artificial mixtures demonstrated that at near equal proportions, *WG-FAST* can place a sample in the wrong location (Additional file 10). When a long branch is observed on a placed sample, other evidence must be considered when evaluating the quality of a placement, including the number of positions required for accurate placement, determined by subsampling, in that region of the phylogeny. Removing homoplasious SNPs has the potential to resolve the mixture into dominant and subdominant strains. For each dataset studied, a similar analysis should be conducted in order to understand the limits of the placement method. To determine the fewest number of reads and reference positions that still result in accurate phylogenetic placement, a subsampling approach was employed. The subsampling experiments based on subsampled SNPs demonstrated that a minimum of 100 reference positions must be called in the case of the *E. coli* dataset used in this study to accurately genotype unknowns ≥95 % of the time. However, the region of the tree where the unknown falls can drastically affect the number of required positions, which can be greater than 9,500 (approximately 0.002× genome coverage). There was a strong correlation with the number of positions required for accurate placement and the topology of the tree. In general, nodes that were filled with closely related isolates required only approximately 100 positions for accurate placement, while nodes containing isolates with long branch lengths required far more positions to be called for accurate placement. The sequence analysis of additional diverse isolates will help fill in blank regions in the tree and create a reference phylogeny that will be better able to place unknown isolates at very low read coverage.

When compiling a reference database for a pathogen of interest, the clonality of an organism should be considered. For highly recombinant pathogens, such as *Burkholderia pseudomallei*, *WG-FAST* analysis may require additional positions to be called in order to separate the clonal signal from the recombinant signal. For highly clonal pathogens, the issue becomes the relative lack of polymorphisms in the dataset. For example, only 2,298 SNPs are able to describe the global phylogenetic diversity in *Yersinia pestis* [47], which will require more sequence reads to accurately place an unknown due to the reduced size of the available SNP search space.

The large sequence datasets that are now available to most researchers have presented new problems, both computationally and methodologically, for the analysis of new isolates. *WG-FAST* presents a method to characterize new isolates in the context of a reference population. The applications to this method include assigning isolates to known outbreaks, as described in this study, typing unknown isolates to specific phylogenetic lineages, and may provide the resolution to resolve transmission routes, although additional experimentation is required before this is verified. As sequence data, both single isolate and metagenomic, become more commonplace, methods that scale linearly with huge datasets, such as *WG-FAST*, will become critical for the analysis of clinical pathogens.

## Conclusions

In this study, we demonstrate how *WG-FAST* can be used to genotype isolates at the strain level from complex samples using low levels of sequence data obtained from metagenomics studies. While *WG-FAST* can also be used in conjunction with single isolate genomics datasets, it is especially powerful when analyzing low coverage datasets. In addition to genotyping, *WG-FAST* performs statistical analysis to help assess the quality of an unknown placement. We demonstrate that in *E. coli*, *WG-FAST* can be used to genotype from metagenomic datasets, place samples accurately at extremely low reference genome coverage, and provide a confidence landscape when assessing placement confidence. As reference databases and sequence datasets become more complex, methods such as *WG-FAST* are required for strain-level genotyping.

## Additional files

Additional file 1:
**A Newick-formatted phylogenetic tree for the 255** ***E. coli*** **genomes analyzed in this study.**
Additional file 2:
**Reference** ***Escherichia coli*** **genomes used in this study.**
Additional file 3:
**Is a NASP-formatted SNP matrix compatible with** ***WG-FAST*** **; this matrix includes >200,000 SNPs in 255** ***E. coli*** **genomes.**
Additional file 4:
**Information for genomes used for read subsampling experiments.**
Additional file 5:
**Information regarding** ***E. coli*** **metagenomic samples [** 34 **] processed with** ***WG-FAST*** **.**
Additional file 6:
**Read information for** ***in silico*** **mixtures generated in this study.**
Additional file 7:
**Percent error across the reference** ***E. coli*** **genome TY-2482.** Reads were mapped to the chromosome and calls were determined at each position in the reference genome. The dominant calls were removed and all other calls were assumed to be error. Positions with differing levels of error were binned and plotted. Additional file 8:
**A maximum likelihood phylogeny of all considered** ***E. coli*** **genomes, inferred from >225,000 single nucleotide polymorphisms (SNPs) with RAxML v8 [** 23 **].** Taxa names are assembly IDs from GenBank and correspond to specific strains (Additional file 2). Additional file 9:
**A comparison of patristic distance between subsampled genomes with genomes using all available data.** Reads were randomly sampled at various depths, re-inserted into the phylogeny, and the patristic distance was calculated between the subsampled placement and the correct placement. This procedure was performed 1,000 times and the resulting patristic distances were plotted. Additional file 10:
**Phylogenetic trees demonstrating the effect of mixtures on phylogenetic placement with** ***WG-FAST*** **.** *Escherichia coli* isolates 8624 and C227-11 were mixed *in silico* at different read mixtures in a total of 10,000 reads. Red isolates indicate the placements with *WG-FAST*. Each read mixture was separately sampled and placed 10 times. The reference genome is *E. coli* K-12 W3110. Additional file 11:
**A scatter plot demonstrating the effect of SNP sampling on insertion likelihood values produced by RAxML.** For each of 100 replicates, reads were randomly selected from *E. coli* C227-11 and processed with *WG-FAST*. The insertion likelihood values from RAxML v8 were then plotted at each sampling depth. This chart demonstrates that the insertion likelihood values increase with increased sampling depth. Additional file 12:
**A scatter plot demonstrating the effect of increasing read depth on** ***P*** **values produced by the** ***WG-FAST*** **subsampling routine.** In each case, raw reads were randomly sampled from *E. coli* 8624 and placed with *WG-FAST*, using 50 iterations. Average *P* values were plotted. Error bars indicate standard deviation from the mean, with lower error bars capped at 0.
