# Supplementary material for: Phylogenetically typing bacterial strains from partial SNP genotypes observed from direct sequencing of clinical specimen metagenomic data
Source: Genome Med. 2015 Jun 9;7:52. doi: 10.1186/s13073-015-0176-9 (PMC4487561; doi:10.1186/s13073-015-0176-9)
Supplement: Additional file 2: — Reference Escherichia coli genomes used in this study. [file 13073_2015_176_MOESM2_ESM.pdf]

Genome name	Assembly name
W3110	ASM1024v1
SE11	ASM1038v1
SE15	ASM1048v1
UTI89	ASM1326v1
536	ASM1330v1
KO11FL	ASM14785v3
APEC_01	ASM1484v1
B088	ASM16315v1
B185	ASM16317v1
B354	ASM16319v1
MS_198-1	ASM16419v1
MS_84-1	ASM16421v1
MS_115-1	ASM16423v1
MS_146-1	ASM16427v1
MS_69-1	ASM16431v1
MS_187-1	ASM16433v1
MS_21-1	ASM16435v1
MS_117-3	ASM16437v1
MS_153-1	ASM16443v1
MS_78-1	ASM16445v1
MS_116-1	ASM16447v1
MS_175-1	ASM16451v1
MS_196-1	ASM16455v1
MS_185-1	ASM16457v1
MS_57-2	ASM16461v1
1827-70	ASM16565v1
TW10598	ASM16653v2
TW10828	ASM16657v2
TW11681	ASM16659v2
TW14425	ASM16661v2
B7A	ASM16781v1
E110019	ASM16787v1
53638	ASM16791v2
101-1	ASM16809v1
H736	ASM17653v2
M718	ASM17657v2
TA206	ASM17659v2
TA143	ASM17661v2
TA280	ASM17665v2
H591	ASM17667v2
H299	ASM17669v2
E24377A	ASM1774v1
HS	ASM1776v1
MS_79-10	ASM17909v1
MS_145-7	ASM17911v1
3431	ASM18476v2
G5101	ASM18728v4
3256-97	ASM18734v2
USDA_5905	ASM18736v2
LT-68	ASM18881v2
OK1357	ASM18885v2
E1167	ASM19079v1
E1520	ASM19081v1
E482	ASM19083v1
H120	ASM19085v1
TA007	ASM19097v1
H489	ASM19101v1
ATCC_8739	ASM1938v1
WV_060327	ASM19397v2
EC4100B	ASM19399v2
97.0246	ASM19421v2
97.0259	ASM19423v2
95.0941	ASM19425v1
97.0264	ASM19429v1
JB1-95	ASM19435v2
1.2264	ASM19441v2
99.0741	ASM19443v2
9.1649	ASM19447v1
2.4168	ASM19455v2
TW07793	ASM19468v2
B41	ASM19470v2
SMS-3-5	ASM1964v1
H10407	ASM21047v1
AA86	ASM21139v1
UMNK88	ASM21271v2
NA114	ASM21476v2
5.0588	ASM21514v2
3.2608	ASM21520v2
9.0111	ASM21526v2
3.3884	ASM21528v2
PCN033	ASM21951v2
STEC_B2F1	ASM22502v2
STEC_C165-02	ASM22504v2
3030-1	ASM22508v2
STEC_94C	ASM22510v2
STEC_DG131-3	ASM22512v2
STEC_EH250	ASM22514v2
G58-1	ASM22516v2
STEC_MHI813	ASM22520v2
STEC_S1191	ASM22522v2
TX1999	ASM22524v2
BL21(DE3)	ASM2266v1
O7:K1_CE10	ASM22762v1
cloneA_i1	ASM23367v2
O113:H21_CL-3	ASM23423v2
O121:H19_MT#2	ASM23427v2
O145:H28_4865/96	ASM23431v2
BL21-Gold(DE3)pLysS	ASM2366v1
DEC1D	ASM24911v2
DEC2A	ASM24915v2
DEC5E	ASM24957v2
DEC6B	ASM24961v2
DEC6D	ASM24965v2
DEC7B	ASM24971v2
DEC9E	ASM24997v2
DEC10F	ASM25009v2
DEC11A	ASM25011v2
DEC12C	ASM25025v2
DEC13A	ASM25031v2
DEC14A	ASM25041v2
DEC14C	ASM25045v2
DEC14D	ASM25047v2
DEC15A	ASM25049v2
SCI-07	ASM25280v2
P12b	ASM25727v1
W26	ASM25822v1
ON2010	ASM25863v1
O32:H37_P4	ASM25942v1
IAI1	ASM2626v1
O103:H25_CVM9340	ASM26389v1
O26:H11_CVM10026	ASM26403v1
KD1	ASM26409v1
541-15	ASM26411v1
75	ASM26415v1
541-1	ASM26421v1
CUMT8	ASM26423v1
O127:H6_E2348/69	ASM2654v1
C342-62	ASM26820v1
042	ASM2712v1
STEC_O31	ASM28177v1
LF82	ASM28449v1
J96	ASM29577v2
0.1288	ASM30325v1
EC1864	ASM30341v1
N1	ASM30363v1
07798	ASM30365v1
ARS4.2123	ASM30409v1
TW00353	ASM30411v1
3006	ASM30413v1
8.0566	ASM30517v1
chi7122	ASM30720v1
E2265	E2265_1
AI27	ECAI27.hybrid.1
AB42410445-isolate1	E_coliAB42410445-isolate1-1.0
NCCP15657	EscCol1.0
4_1_47FAA	Esch_coli_4_1_47FAA_V1
B093	Esch_coli_B093_V1
B799	Esch_coli_B799_V1
O104:H4_C227-11	Esch_coli_C227-11_V2
E101	Esch_coli_E101_V1
H494	Esch_coli_H494_V1
KTE100	Esch_coli_KTE100_V1
KTE102	Esch_coli_KTE102_V1
KTE103	Esch_coli_KTE103_V1
KTE107	Esch_coli_KTE107_V1
KTE108	Esch_coli_KTE108_V1
KTE10	Esch_coli_KTE10_V1
KTE111	Esch_coli_KTE111_V1
KTE115	Esch_coli_KTE115_V1
KTE119	Esch_coli_KTE119_V1
KTE120	Esch_coli_KTE120_V1
KTE121	Esch_coli_KTE121_V1
KTE122	Esch_coli_KTE122_V1
KTE134	Esch_coli_KTE134_V1
KTE135	Esch_coli_KTE135_V1
KTE136	Esch_coli_KTE136_V1
KTE13	Esch_coli_KTE13_V1
KTE142	Esch_coli_KTE142_V1
KTE144	Esch_coli_KTE144_V1
KTE147	Esch_coli_KTE147_V1
KTE14	Esch_coli_KTE14_V1
KTE154	Esch_coli_KTE154_V1
KTE155	Esch_coli_KTE155_V1
KTE156	Esch_coli_KTE156_V1
KTE158	Esch_coli_KTE158_V1
KTE161	Esch_coli_KTE161_V1
KTE165	Esch_coli_KTE165_V1
KTE171	Esch_coli_KTE171_V1
KTE172	Esch_coli_KTE172_V1
KTE178	Esch_coli_KTE178_V1
KTE17	Esch_coli_KTE17_V1
KTE181	Esch_coli_KTE181_V1
KTE182	Esch_coli_KTE182_V1
KTE184	Esch_coli_KTE184_V1
KTE185	Esch_coli_KTE185_V1
KTE186	Esch_coli_KTE186_V1
KTE187	Esch_coli_KTE187_V1
KTE189	Esch_coli_KTE189_V1
KTE190	Esch_coli_KTE190_V1
KTE191	Esch_coli_KTE191_V1
KTE192	Esch_coli_KTE192_V1
KTE193	Esch_coli_KTE193_V1
KTE195	Esch_coli_KTE195_V1
KTE196	Esch_coli_KTE196_V1
KTE197	Esch_coli_KTE197_V1
KTE19	Esch_coli_KTE19_V1
KTE202	Esch_coli_KTE202_V1
KTE206	Esch_coli_KTE206_V1
KTE209	Esch_coli_KTE209_V1
KTE20	Esch_coli_KTE20_V1
KTE210	Esch_coli_KTE210_V1
KTE212	Esch_coli_KTE212_V1
KTE213	Esch_coli_KTE213_V1
KTE214	Esch_coli_KTE214_V1
KTE215	Esch_coli_KTE215_V1
KTE211	Esch_coli_KTE221_V1
KTE222	Esch_coli_KTE222_V1
KTE227	Esch_coli_KTE227_V1
KTE228	Esch_coli_KTE228_V1
KTE22	Esch_coli_KTE22_V1
KTE233	Esch_coli_KTE233_V1
KTE234	Esch_coli_KTE234_V1
KTE24	Esch_coli_KTE24_V1
KTE26	Esch_coli_KTE26_V1
KTE28	Esch_coli_KTE28_V1
KTE29	Esch_coli_KTE29_V1
KTE35	Esch_coli_KTE35_V1
KTE37	Esch_coli_KTE37_V1
KTE40	Esch_coli_KTE40_V1
KTE41	Esch_coli_KTE41_V1
KTE42	Esch_coli_KTE42_V1
KTE43	Esch_coli_KTE43_V1
KTE44	Esch_coli_KTE44_V1
KTE46	Esch_coli_KTE46_V1
KTE47	Esch_coli_KTE47_V1
KTE48	Esch_coli_KTE48_V1
KTE4	Esch_coli_KTE4_V1
KTE50	Esch_coli_KTE50_V1
KTE54	Esch_coli_KTE54_V1
KTE56	Esch_coli_KTE56_V1
KTE57	Esch_coli_KTE57_V1
KTE60	Esch_coli_KTE60_V1
KTE61	Esch_coli_KTE61_V1
KTE63	Esch_coli_KTE63_V1
KTE64	Esch_coli_KTE64_V1
KTE67	Esch_coli_KTE67_V1
KTE68	Esch_coli_KTE68_V1
KTE73	Esch_coli_KTE73_V1
KTE75	Esch_coli_KTE75_V1
KTE76	Esch_coli_KTE76_V1
KTE77	Esch_coli_KTE77_V1
KTE79	Esch_coli_KTE79_V1
KTE7	Esch_coli_KTE7_V1
KTE80	Esch_coli_KTE80_V1
KTE81	Esch_coli_KTE81_V1
KTE84	Esch_coli_KTE84_V1
KTE98	Esch_coli_KTE98_V1
KTE9	Esch_coli_KTE9_V1
M919	Esch_coli_M919_V2
SWW33	Esch_coli_SWW33_V1
TA124	Esch_coli_TA124_V1
NCCP15647	NCCP15647_1.0
NCCP15658	NCCP15658v1.0
TW10509	TW10509
LCT-EC52	genome_assembly_of_LCT-EC52_strain_of_E.coli
