# Supplementary material for: Phylogenetically typing bacterial strains from partial SNP genotypes observed from direct sequencing of clinical specimen metagenomic data
Source: Genome Med. 2015 Jun 9;7:52. doi: 10.1186/s13073-015-0176-9 (PMC4487561; doi:10.1186/s13073-015-0176-9)
Supplement: Additional file 4: — Information for genomes used for read subsampling experiments. [file 13073_2015_176_MOESM4_ESM.pdf]

Genome	Serotype	Pathovar	Phylogroup	SRA accession	Sequencing Platform
C227-11	O104:H4	EAEC/STEC	B1	SRR341579	HiSeq
86-24	0157:H7	STEC	E	SRR1639082	HiSeq
C844-97	O157:H45	aEPEC	B2	SRR1772997	HiSeq
sakai	O157:H7	STEC	E	SRR587217	MiSeq
C260-92	O127:K-:H40	aEPEC	A	SRR1773018	HiSeq
KTE9	unknown	N/A	D	SRR640937	HiSeq

read length	average positions for accurate placement > 95%	read coverage for
100	979	<0.01x
100	932	<0.01x
100	1062	<0.01x
250	361	<0.01x
100	73664	0.42x
100	2663	0.14x
