# Supplementary figures and images for: Phylogenetically typing bacterial strains from partial SNP genotypes observed from direct sequencing of clinical specimen metagenomic data
Source: Genome Med. 2015 Jun 9;7:52. doi: 10.1186/s13073-015-0176-9 (PMC4487561; doi:10.1186/s13073-015-0176-9)

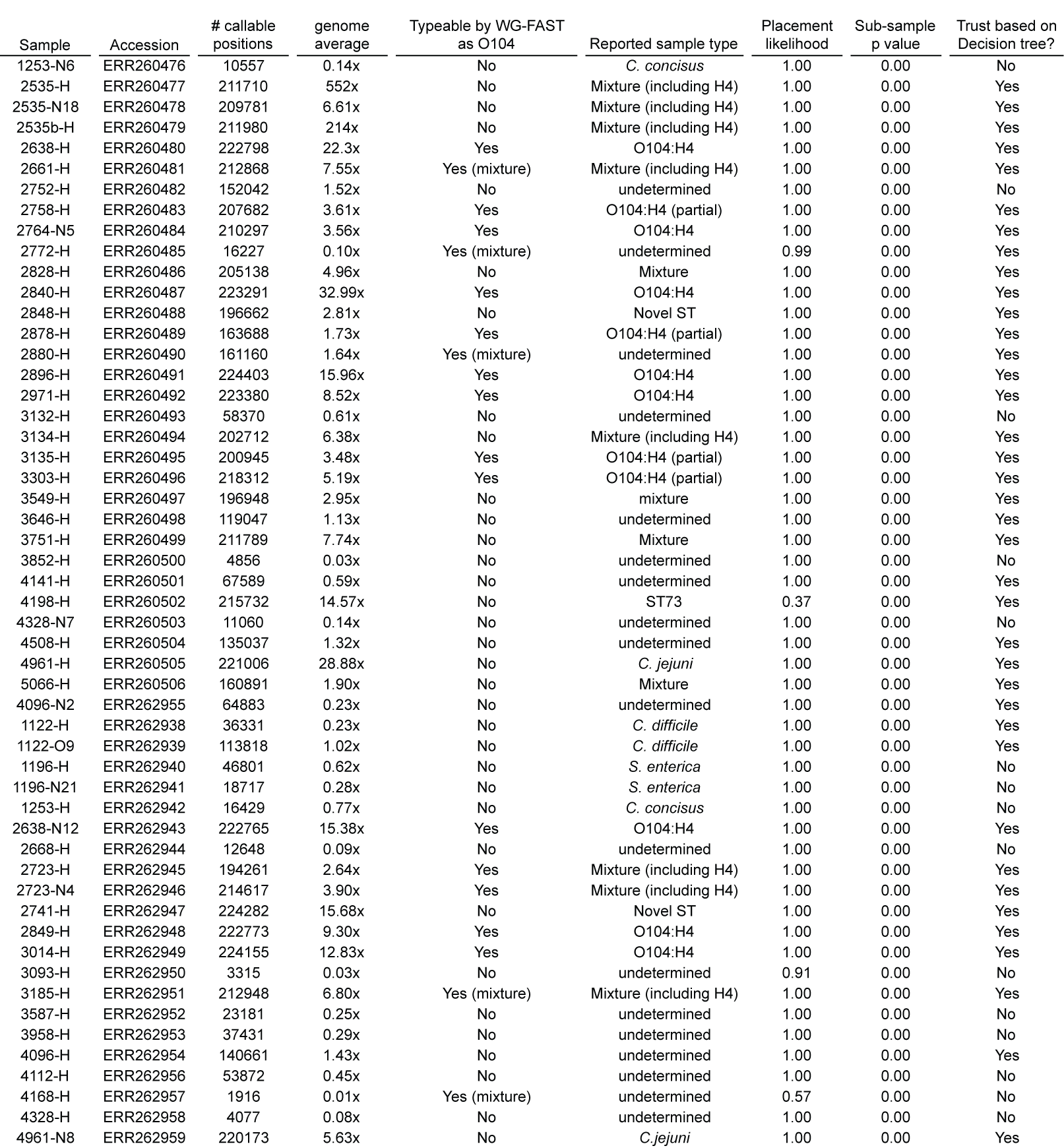

Supplement: Additional file 5: — Information regarding E. coli metagenomic samples [ 34 ] processed with WG-FAST . [file 13073_2015_176_MOESM5_ESM.png]

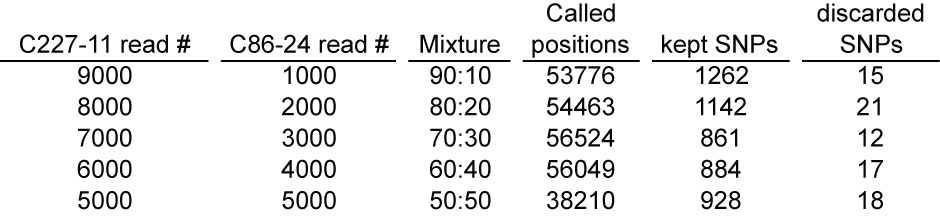

Supplement: Additional file 6: — Read information for in silico mixtures generated in this study. [file 13073_2015_176_MOESM6_ESM.png]

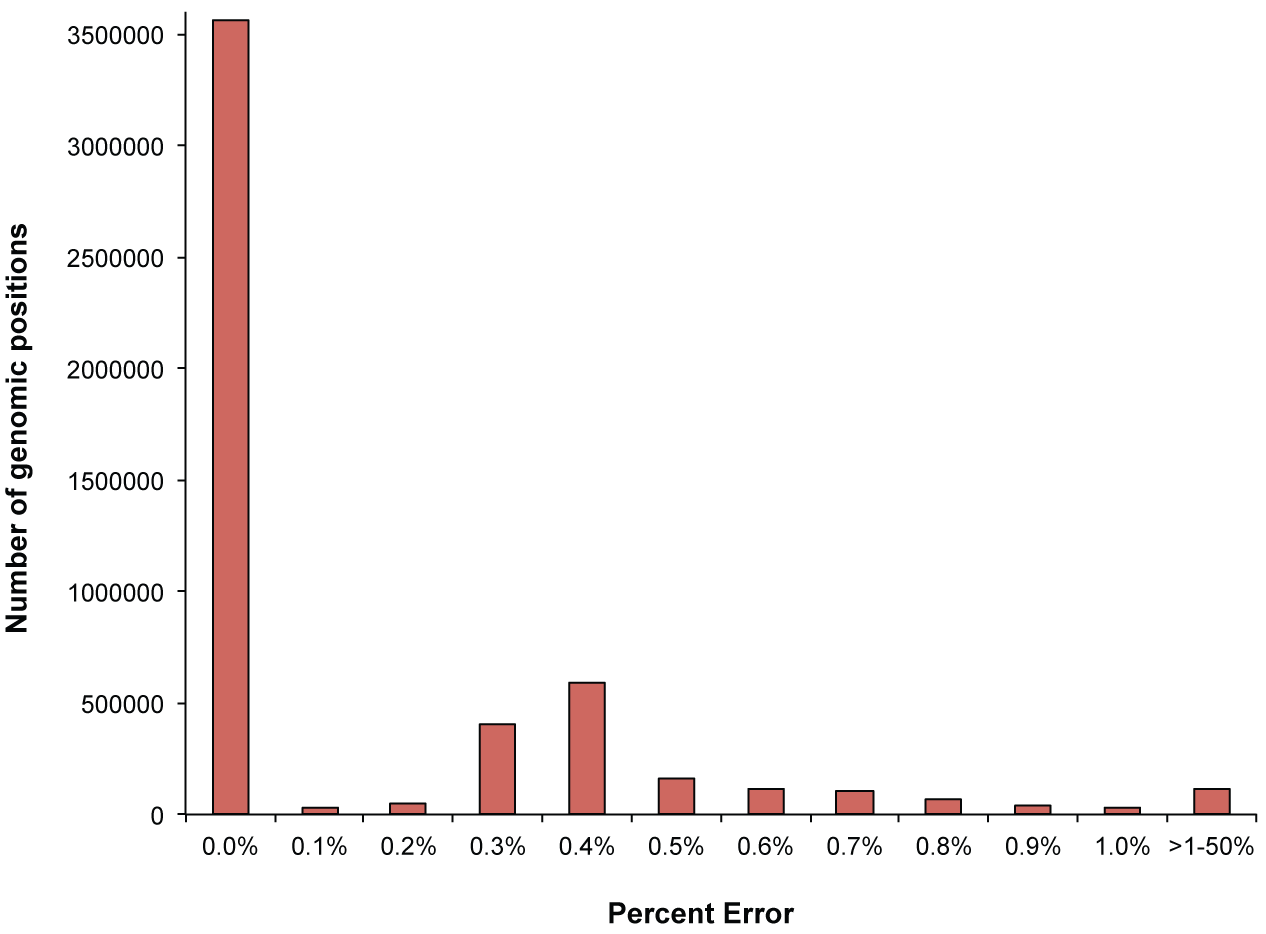

Supplement: Additional file 7: — Percent error across the reference E. coli genome TY-2482. Reads were mapped to the chromosome and calls were determined at each position in the reference genome. The dominant calls were removed and all other calls were assumed to be error. Positions with differing levels of error were binned and plotted. [file 13073_2015_176_MOESM7_ESM.png]

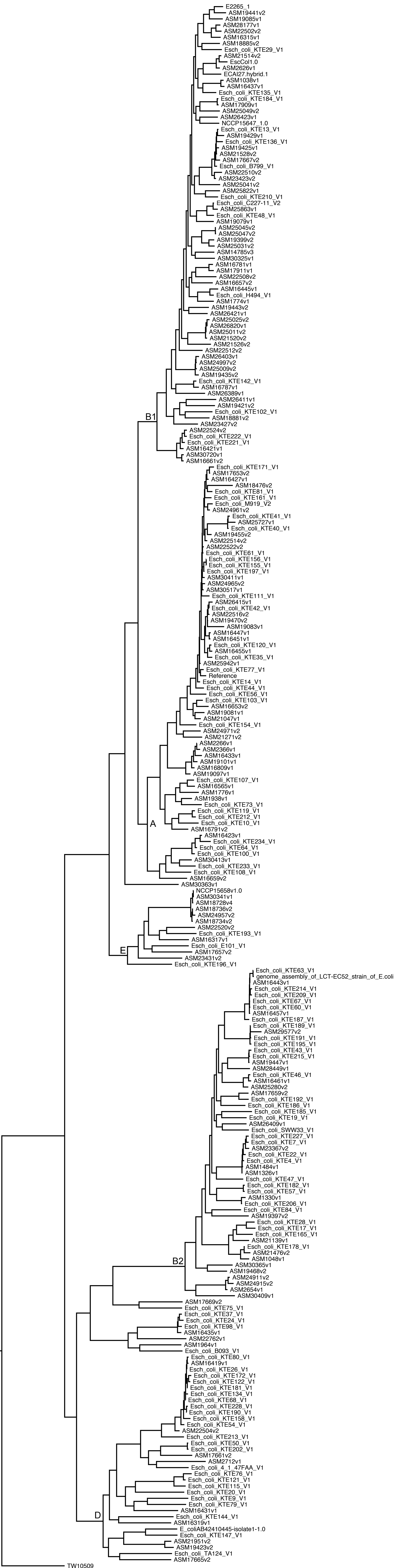

Supplement: Additional file 8: — A maximum likelihood phylogeny of all considered E. coli genomes, inferred from >225,000 single nucleotide polymorphisms (SNPs) with RAxML v8 [ 23 ]. Taxa names are assembly IDs from GenBank and correspond to specific strains (Additional file 2). [file 13073_2015_176_MOESM8_ESM.pdf]

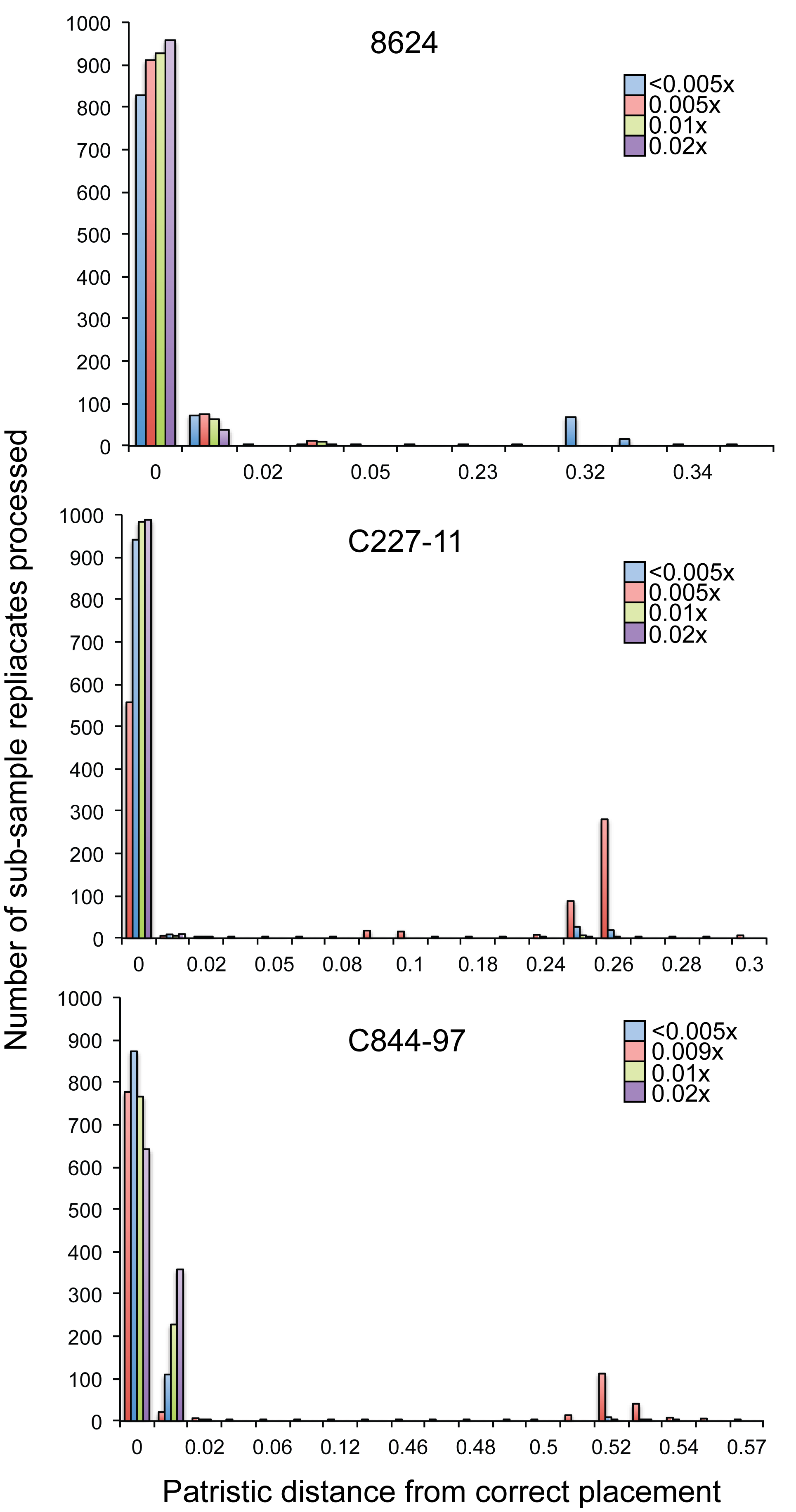

Supplement: Additional file 9: — A comparison of patristic distance between subsampled genomes with genomes using all available data. Reads were randomly sampled at various depths, re-inserted into the phylogeny, and the patristic distance was calculated between the subsampled placement and the correct placement. This procedure was performed 1,000 times and the resulting patristic distances were plotted. [file 13073_2015_176_MOESM9_ESM.png]

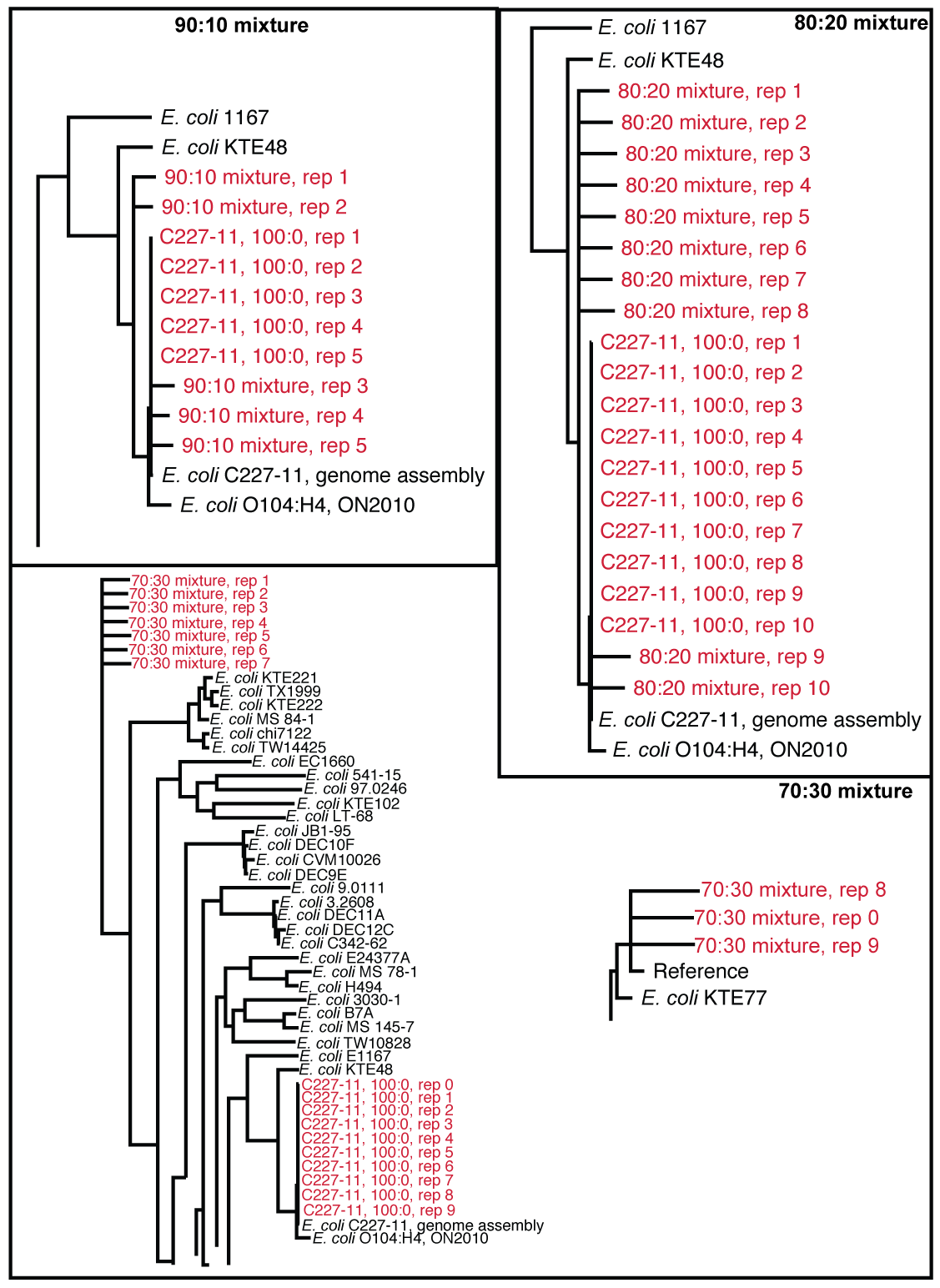

Supplement: Additional file 10: — Phylogenetic trees demonstrating the effect of mixtures on phylogenetic placement with WG-FAST . Escherichia coli isolates 8624 and C227-11 were mixed in silico at different read mixtures in a total of 10,000 reads. Red isolates indicate the placements with WG-FAST. Each read mixture was separately sampled and placed 10 times. The reference genome is E. coli K-12 W3110. [file 13073_2015_176_MOESM10_ESM.png]

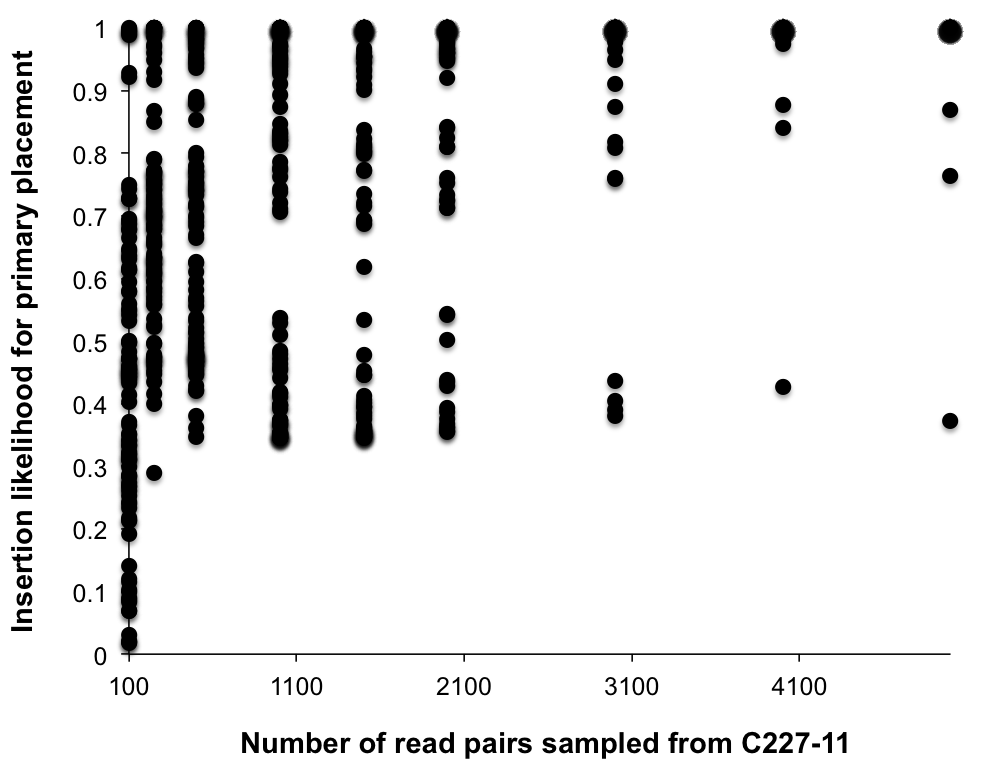

Supplement: Additional file 11: — A scatter plot demonstrating the effect of SNP sampling on insertion likelihood values produced by RAxML. For each of 100 replicates, reads were randomly selected from E. coli C227-11 and processed with WG-FAST. The insertion likelihood values from RAxML v8 were then plotted at each sampling depth. This chart demonstrates that the insertion likelihood values increase with increased sampling depth. [file 13073_2015_176_MOESM11_ESM.png]

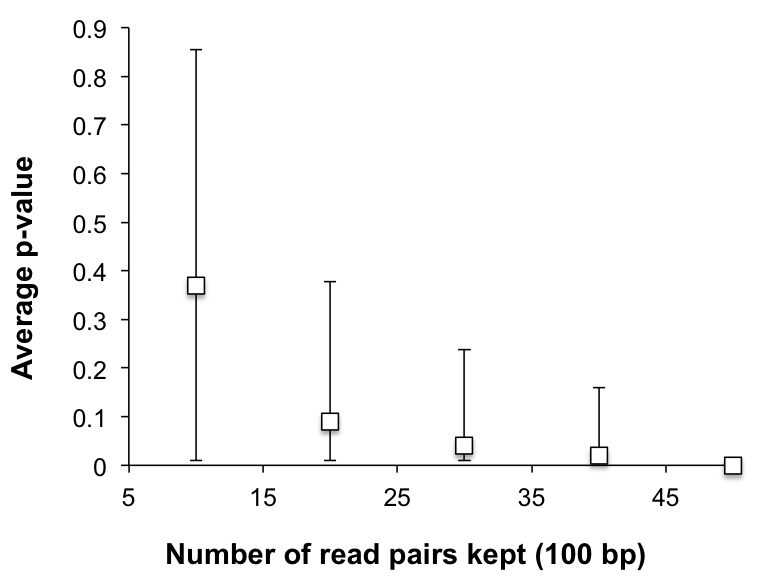

Supplement: Additional file 12: — A scatter plot demonstrating the effect of increasing read depth on P values produced by the WG-FAST subsampling routine. In each case, raw reads were randomly sampled from E. coli 8624 and placed with WG-FAST, using 50 iterations. Average P values were plotted. Error bars indicate standard deviation from the mean, with lower error bars capped at 0. [file 13073_2015_176_MOESM12_ESM.png]
